# Structure and Hirshfeld surface analysis of the salt *N*,*N*,*N*-trimethyl-1-(4-vinyl­phen­yl)methanaminium 4-vinyl­benzene­sulfonate

**DOI:** 10.1107/S2056989019007758

**Published:** 2019-06-04

**Authors:** C. John McAdam, Lyall R. Hanton, Stephen C. Moratti, Jim Simpson, Ravindra N. Wickramasinhage

**Affiliations:** a Department of Chemistry, University of Otago, PO Box 56, Dunedin, New Zealand

**Keywords:** crystal structure, *N*,*N*,*N*-trimethyl-1-(4-vinyl­phen­yl)methanaminium cation, 4-vinyl­benzene­sulfonate anion, hydrogen bonds, Hirshfeld surface analysis

## Abstract

In the title compound, the asymmetric unit comprises an *N*,*N*,*N*-trimethyl-1-(4-vinyl­phen­yl)methanaminium cation and a 4-vinyl­benzene­sulfonate anion, C_12_H_18_N^+^·C_8_H_7_O_3_S^−^. The salt has a polymerizable vinyl group attached to both the cation and the anion. The methanaminium and vinyl substituents on the benzene ring of the cation subtend angles of 86.6 (3) and 10.5 (9)° to the ring plane, while the anion is planar excluding the sulfonate O atoms. The vinyl substituent on the benzene ring of the cation is disordered over two sites with a refined occupancy ratio of 0.542 (11):0.458 (11). In the crystal, C—H⋯O hydrogen bonds dominate the packing and combine with a C—H⋯π(ring) contact to stack the cations and anions along the *a*-axis direction. Hirshfeld surface analysis of the salt and of the individual cation and anion components is also reported.

## Abstract 2

The mol­ecular and crystal structure of the salt *N*,*N*,*N*-trimethyl-1-(4-vinyl­phen­yl)methanaminium 4-vinyl­benzene­sulfonate is reported. A Hirshfeld surface analysis of the salt and its individual components is also presented.

## Chemical context   

Hydro­gels continue to be the subject of intense study, particularly with regard to biomedical applications and new technologies (Van Vlierberghe *et al.*, 2011 ▸; Sun *et al.*, 2015 ▸; Goswami *et al.*, 2017 ▸; Pushparajan *et al.*, 2018 ▸). Limiting development has been the poor mechanical strength of conventional hydro­gel formulations. Numerous strategies, singly and in combination, have been utilized in efforts to improve toughness and stretchability, and the results have been extensively reviewed (Naficy *et al.*, 2011 ▸; Peak *et al.*, 2013 ▸; Zhao, 2014 ▸). Our current approach is to build in capacity for self-healing, and exploits polyampholytes (Zurick & Bernards, 2014 ▸), polymers formed from the covalent cross-linking of mixed cationic and anionic monomers. The title compound is one such set of ion-pair co-monomers, simply prepared from commercially available tri­methyl­ammonium cation and sulfonate anion salts.
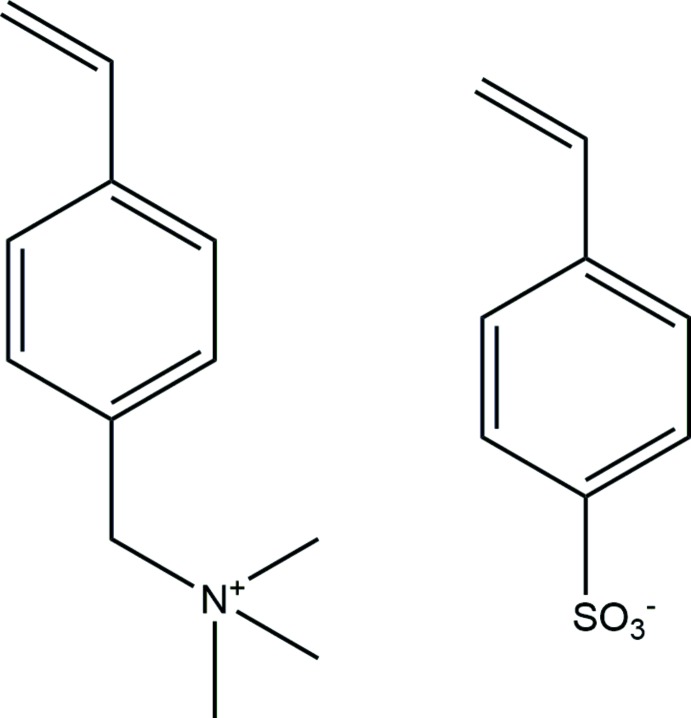


## Structural commentary   

**Figure 1:**
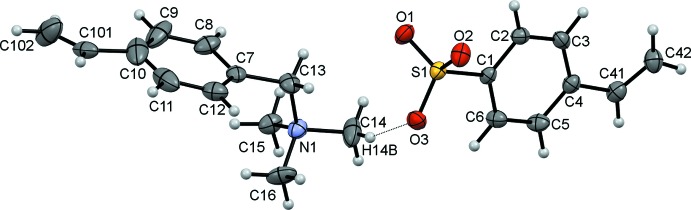
The asymmetric unit of the title compound showing the atom numbering with ellipsoids drawn at the 50% probability level. The C—H⋯O hydrogen bond linking the two components is drawn as a dotted black line. For clarity, only the major disorder component of the vinyl substituent on the benzene ring of the cation is shown.

The asymmetric unit of the title salt, (I), comprises an *N*,*N*,*N*-trimethyl-1-(4-vinyl­phen­yl)methanaminium cation and a 4-vinyl­benzene­sulfonate anion, linked by a C14—H14*B*⋯O3 hydrogen bond (Table 1 ▸) between a methyl group of the tri­methyl­methanaminium unit and a sulfonate oxygen, Fig. 1 ▸. The vinyl substituent on the benzene ring of the cation is disordered over two sites with a refined occupancy ratio of 0.542 (11):0.458 (11). In the cation, the C7/C13/N1 and C10/C101/C102 planes of the methanaminium and major vinyl substituents on the benzene ring subtend angles of 86.6 (3) and 10.5 (9)°, respectively, to the ring plane. In contrast, excluding the sulfonate O atoms, the S and ordered vinyl substituents lie close to the benzene ring plane in the anion with an r.m.s. deviation of 0.0753 Å from the S1/C1–C6/C41/C42 plane.

## Supra­molecular features   

**Figure 2:**
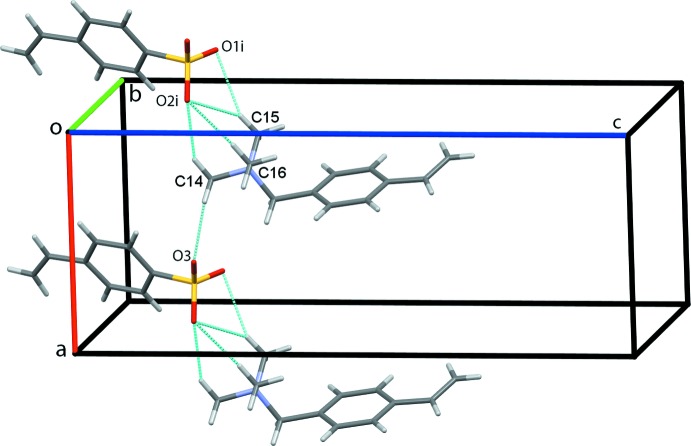
Chains of cations and anions of (I) along the *a* axis. Hydrogen bonds are shown as cyan dotted lines [symmetry code: (i) *x* − 1, *y*, *z*].

**Figure 3:**
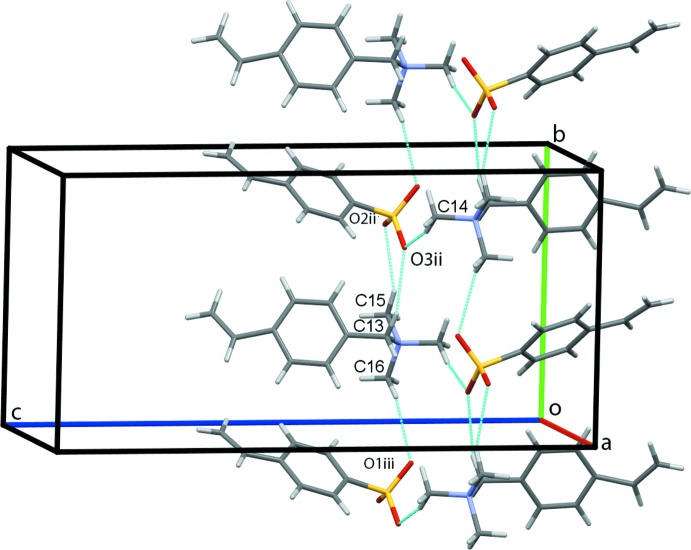
Double chains of cation–anion dimers along *b*. Hydrogen bonds are shown as cyan dotted lines [symmetry codes: (ii) 1 − *x*, 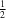 + *y*, 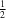 − *z*; (iii) 1 − *x*, −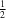 + *y*, 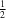 − *z*].

**Figure 4:**
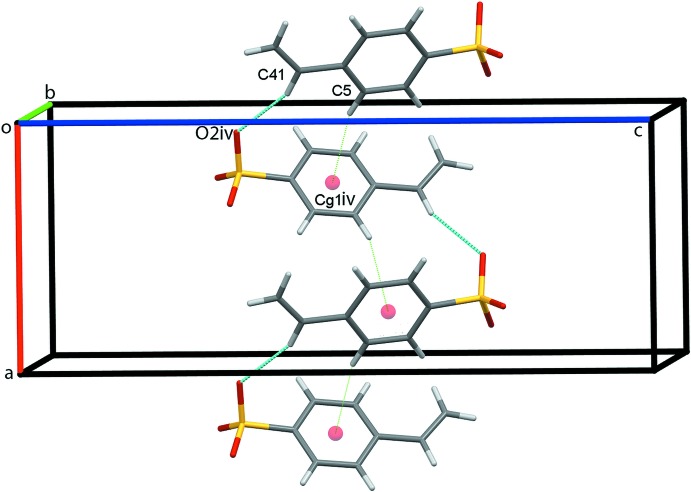
Chains of anions along *a*. Hydrogen bonds and C—H⋯π inter­actions are shown as cyan and green dotted lines, respectively [symmetry code: (iv) *x* − 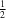, 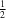 − *y*, −*z*].

**Figure 5:**
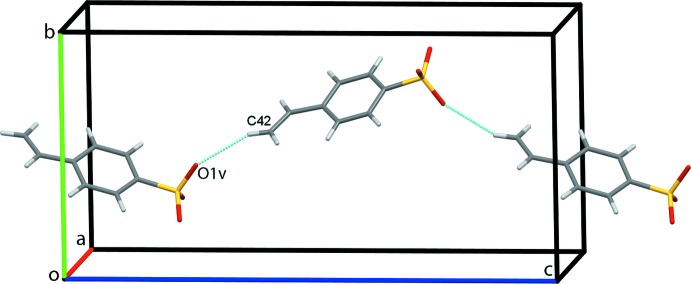
Zigzag chains of anions along *c*. Hydrogen bonds are shown as cyan dotted lines [symmetry code: (v) 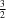 − *x*, 1 − *y*, *z* − 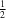].

**Figure 6:**
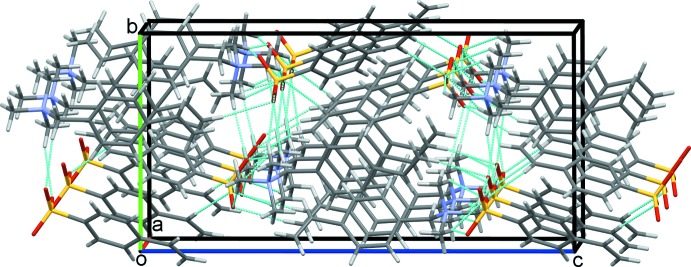
Overall packing for (I) viewed along the *a*-axis direction.

Packing in this salt is dominated by an extensive number of C—H⋯O hydrogen bonds, Table 1 ▸. O2 acts as a trifurcated acceptor forming C14—H14*A*⋯O2^i^, C15—H15*A*⋯O2^i^ and C16—H16*C*⋯O2^i^ hydrogen bonds [symmetry code: (i) *x* − 1, *y*, *z*]. C14 and C15 are bifurcated donors with the C15—H15*A*⋯O1^i^ and C15—H15*A*⋯O2^i^ contacts forming 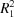
(4) ring motifs. C14—H14*B*⋯O3 contacts link the cation–anion pairs into chains along the *a-*axis direction, Fig. 2 ▸. Cation–anion dimers are generated by C13—H13*B*⋯O3^ii^ and C15—H15*B*⋯O2^ii^ contacts with adjacent dimers linked into columns along *b* by C16—H16*B*⋯O1^iii^ hydrogen bonds [symmetry codes: (ii) 1 − *x*, 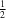 + *y*, 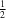 − *z*; (iii) 1 − *x*, −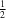 + *y*, 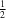 − *z*]. Additional C14—H14*B*⋯O3 hydrogen bonds form double columns along *b* with the vinyl substituents of the proximate cations and anions pointing in opposite directions, Fig. 3 ▸. Chains of anions form along *a* through C41—H41⋯O2^iv^ hydrogen bonds augmented by C5—H5⋯C*g*1^iv^ contacts, Fig. 4 ▸ [symmetry code: (iv) *x* − 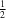, 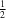 − *y*, −*z*]. Finally, weak C42—H42*B*⋯O1^v^ hydrogen bonds link the anions in a head-to-tail fashion into zigzag chains along *c*, Fig. 5 ▸ [symmetry code: (v) 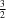 − *x*, 1 − *y*, *z* − 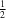]. This extensive series of contact combines to assemble an extended network structure with the cations and anions stacked along the *a*-axis direction, Fig. 6 ▸.

## Hirshfeld surface analysis   

**Figure 7:**
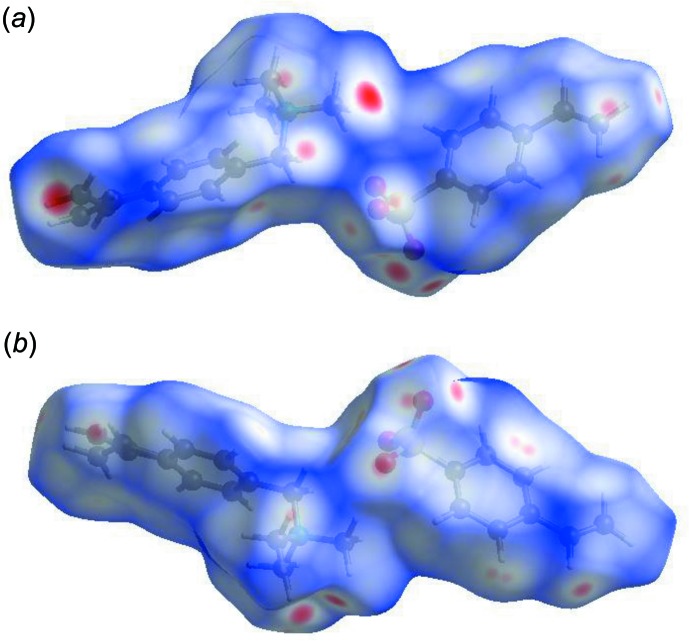
Hirshfeld surfaces of (1) viewed for opposite faces of the salt.

**Figure 8:**
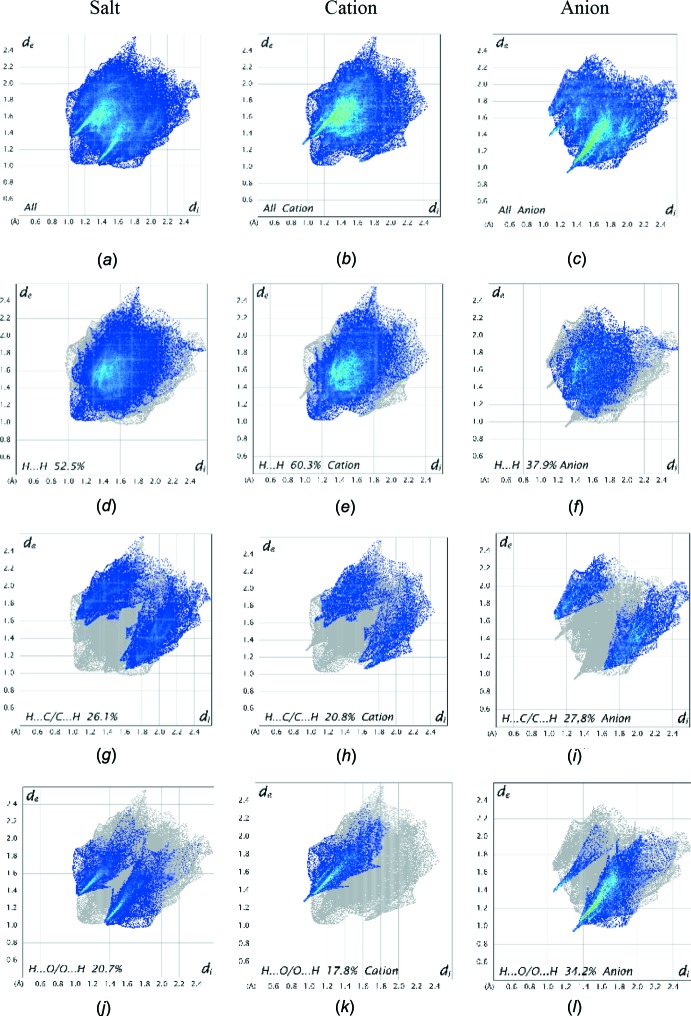
Full two-dimensional fingerprint plots for the salt (*a*), cation (*b*) and anion (*c*) together with (*d*)–(*l*) separate principal contact types for the salt, cation and anion systems respectively. These are found to be H⋯H, H⋯C/C⋯H, and H⋯O/O⋯H contacts.

Further details of the inter­molecular architecture of this salt were obtained using Hirshfeld surface analysis (Spackman & Jayatilaka, 2009 ▸) with surfaces and two-dimensional fingerprint plots generated by *CrystalExplorer* (Turner *et al.*, 2017 ▸). Hirshfeld surfaces viewed for opposite faces of the complete salt are shown in Fig. 7 ▸. Both disorder components are included in these surface calculations. The red circles on the Hirshfeld surfaces correspond to the numerous C—H⋯O contacts that play a significant role in stabilizing the packing in this structure. Fingerprint plots of the principal contacts on the Hirshfeld surface of the salt are shown in Fig. 8 ▸. These comprise H⋯H, H⋯C/C⋯H, and H⋯O/O⋯H contacts. The much less significant C⋯C and H⋯S/S⋯H contributions are not shown in the figure but are detailed in Table 2 ▸.

It is also instructive to investigate the differences in contacts for the discrete cation and anion components of (I) by recording fingerprint plots of the cation and anion individually. All of the surface contributions for the cation and anion are also shown in Table 2 ▸, with fingerprint plots for principal contacts found in the individual cation and anion also displayed in Fig. 8 ▸. The most notable differences between the values for the salt and its components are that the H⋯H van der Waals inter­actions increase significantly for the cation, while the anion shows considerable increases in the H⋯O/O⋯H and H⋯C/C⋯H contacts. These differences reflect the fact that, whereas the contacts for the cations are limited to cation–anion inter­actions, the anions are also involved in distinct anion–anion contacts, *vide supra*. The C⋯C and H⋯S/S⋯H contributions to all of the surfaces are very weak but are included in Table 2 ▸ for completeness.

## Database survey   

A search of the Cambridge Structural Database (Version 5.40 November 2018 with one update; Groom *et al.*, 2016 ▸) reveals the fact that the salt reported here is quite unusual. Only two structures involving the *N*,*N*,*N*-trimeth­yl(4-vinyl­phen­yl)methyl­ammonium cation acting as counter-ions to poly-molybdate (QAJXEH) and poly-tungstate (QAJXAD) anions were found (Vorotnikov *et al.*, 2015 ▸). Structures of salts of the 4-vinyl­benzene­sulfonate anion are slightly more abundant, with organic methyl­quinolinium (RUMGAJ; Lee *et al.*, 2015 ▸) and 4-{2-[4-(di­methyl­amino)­phen­yl]vin­yl}-1-methyl­pyridinium (SAPDAR; Vijay *et al.*, 2012 ▸) cations and hexa­aqua manganese, cobalt and nickel complex cations (SUVBOA, SUVBUG and SUVCAN; Leonard *et al.*, 1999 ▸).

## Synthesis and crystallization   

The title compound was prepared *via* an argentometric mixing approach (Li *et al.*, 2010 ▸) from the silver salt of 4-vinyl­benzene­sulfonic acid, Ag-VBS (Woeste *et al.*, 1993 ▸; Sikkema *et al.*, 2007 ▸) and (vinyl­benz­yl)tri­methyl­ammonium chloride, VBT-Cl (Sigma Aldrich). A suspension of Ag-VBS in water and equimolar amount of VBT-Cl were stirred 30 minutes. After filtration of the AgCl precipitate, the solution was freeze-dried and the ion-pair co-monomers recrystallized from chloro­form as irregular colourless blocks.

ESI MS +ve (*m*/*z*): 176.14 [C_12_H_18_N]^+^; -ve: 183.01 [C_8_H_7_SO_3_]^−^. ^1^H NMR (400 MHz, DMSO-*d*_6_): 5.95 (*dd*, *J* = 18, 1 Hz, 1H, VBT =CH_2_), 5.38 (*dd*, *J* = 11, 1 Hz, 1H, VBT =CH_2_), 6.80 (*dd*, *J* = 18, 11 Hz, 1H, VBT –CH=), 7.61 & 7.50 [2 × (*d*, *J* = 8 Hz, 2H, VBT benzene H)], 4.51 (*s*, 2H, VBT CH_2_), 4.51 (*s*, 2H, VBT CH_2_), 3.02 (*s*, 9H, VBT CH_3_). 5.84 (*dd*, *J* = 18, 1 Hz, 1H, VBS =CH_2_), 5.27 (*dd*, *J* = 11, 1 Hz, 1H, VBS =CH_2_), 6.73 (*dd*, *J* = 18, 11 Hz, 1H, VBS –CH=), 7.57 & 7.42 [2 × (*d*, *J* = 8 Hz, 2H, VBS benzene H)]

## Refinement   

Crystal data, data collection and structure refinement details are summarized in Table 3 ▸. All H atoms were refined using a riding model with *d*(C—H) = 0.95 Å and *U*_iso_(H) = 1.2*U*_eq_(C) for aromatic and vinyl H atoms, *d*(C—H) = 0.99 Å and *U*_iso_(H) = 1.2*U*_eq_(C) for methyl­ene and *d*(C—H) = 0.98 Å and *U*_iso_(H) = 1.5*U*_eq_(C) for methyl H atoms. The vinyl substituent on the benzene ring of the cation is disordered over two sites (C101=C102 and C103=C104) with a refined occupancy ratio of 0.542 (11):0.458 (11).

## Supplementary Material

Crystal structure: contains datablock(s) I. DOI: 10.1107/S2056989019007758/xi2014sup1.cif

Structure factors: contains datablock(s) I. DOI: 10.1107/S2056989019007758/xi2014Isup2.hkl

Supporting information file. DOI: 10.1107/S2056989019007758/xi2014Isup3.cml

CCDC reference: 1919325

Additional supporting information:  crystallographic information; 3D view; checkCIF report

## Figures and Tables

**Table 1:** Hydrogen-bond geometry (Å, °) *Cg*1 is the centroid of the C1–C6 benzene ring.

*D*—H⋯*A*	*D*—H	H⋯*A*	*D*⋯*A*	*D*—H⋯*A*
C14—H14*B*⋯O3	0.98	2.32	3.264 (5)	161
C14—H14*A*⋯O2^i^	0.98	2.48	3.346 (5)	147
C15—H15*A*⋯O1^i^	0.98	2.63	3.544 (4)	155
C15—H15*A*⋯O2^i^	0.98	2.49	3.348 (4)	147
C13—H13*B*⋯O3^ii^	0.99	2.56	3.466 (5)	152
C15—H15*B*⋯O2^ii^	0.98	2.60	3.477 (4)	149
C16—H16*B*⋯O1^iii^	0.98	2.61	3.365 (4)	134
C16—H16*C*⋯O2^i^	0.98	2.52	3.370 (5)	146
C41—H41⋯O2^iv^	0.95	2.58	3.481 (4)	157
C42—H42*B*⋯O1^v^	0.95	2.63	3.494 (4)	151
C5—H5⋯*Cg*1^iv^	0.95	2.93	3.837 (4)	161

Symmetry codes: (i) 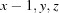; (ii) 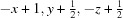; (iii) 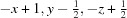; (iv) 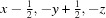; (v) 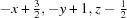.

**Table 2:** Percentage contributions of inter­atomic contacts to the Hirshfeld surface for (I)

Contacts	Included surface area		
	Salt	Cation	Anion
H⋯H	52.5	60.3	37.9
H⋯C/C⋯H	26.1	20.8	27.8
H⋯O/O⋯H	20.7	17.8	34.2
C⋯C	0.5	0.9	0.0
H⋯S/S⋯H	0.1	0.1	0.1

**Table 3:** Experimental details

Crystal data	
Chemical formula	C_12_H_18_N^+^·C_8_H_7_O_3_S^−^
*M*_r_	359.47
Crystal system, space group	Orthorhombic, *P*2_1_2_1_2_1_
Temperature (K)	100
*a*, *b*, *c* (Å)	8.3344 (3), 10.5937 (4), 21.1228 (8)
*V* (Å^3^)	1864.98 (12)
*Z*	4
Radiation type	Cu *K*α
μ (mm^−1^)	1.69
Crystal size (mm)	0.20 × 0.18 × 0.08

Data collection	
Diffractometer	Rigaku Oxford Diffraction SuperNova, Dual, Cu at home/near, Atlas
Absorption correction	Multi-scan (*CrysAlis PRO*; Rigaku OD, 2018 ▸)
*T*_min_, *T*_max_	0.911, 1.000
No. of measured, independent and observed [*I* > 2σ(*I*)] reflections	4767, 3103, 2784
*R*_int_	0.029
(sin θ/λ)_max_ (Å^−1^)	0.620

Refinement	
*R*[*F* ^2^ > 2σ(*F* ^2^)], *wR*(*F* ^2^), *S*	0.040, 0.103, 1.04
No. of reflections	3103
No. of parameters	248
No. of restraints	10
H-atom treatment	H-atom parameters constrained
Δρ_max_, Δρ_min_ (e Å^−3^)	0.37, −0.29
Absolute structure	Flack *x* determined using 870 quotients [(*I* ^+^)−(*I* ^−^)]/[(*I* ^+^)+(*I* ^−^)] (Parsons *et al.*, 2013 ▸)
Absolute structure parameter	−0.040 (19)

Computer programs: *CrysAlis PRO* (Rigaku OD, 2018 ▸), *SHELXT* (Sheldrick, 2015*a* ▸), *SHELXL2018* (Sheldrick, 2015*b* ▸), *TITAN* (Hunter & Simpson, 1999 ▸), *Mercury* (Macrae *et al.*, 2008 ▸), *enCIFer* (Allen *et al.*, 2004 ▸), *PLATON* (Spek, 2009 ▸), *publCIF* (Westrip, 2010 ▸) and *WinGX* (Farrugia, 2012 ▸).

## supplementary crystallographic information

### Crystal data

**Table:**

C_12_H_18_N^+^·C_8_H_7_O_3_S^−^	*D*_x_ = 1.280 Mg m^−^^3^
*M**_r_* = 359.47	Cu *K*α radiation, λ = 1.54184 Å
Orthorhombic, *P*2_1_2_1_2_1_	Cell parameters from 2591 reflections
*a* = 8.3344 (3) Å	θ = 4.2–72.3°
*b* = 10.5937 (4) Å	µ = 1.69 mm^−^^1^
*c* = 21.1228 (8) Å	*T* = 100 K
*V* = 1864.98 (12) Å^3^	Irregular block, colourless
*Z* = 4	0.20 × 0.18 × 0.08 mm
*F*(000) = 768	

### Data collection

**Table:**

Rigaku Oxford Diffraction SuperNova, Dual, Cu at home/near, Atlas diffractometer	3103 independent reflections
Radiation source: micro-focus sealed X-ray tube	2784 reflections with *I* > 2σ(*I*)
Detector resolution: 5.1725 pixels mm^-1^	*R*_int_ = 0.029
ω scans	θ_max_ = 72.8°, θ_min_ = 4.2°
Absorption correction: multi-scan (CrysAlis PRO; Rigaku OD, 2018)	*h* = −6→10
*T*_min_ = 0.911, *T*_max_ = 1.000	*k* = −12→12
4767 measured reflections	*l* = −25→24

### Refinement

**Table:**

Refinement on *F*^2^	Hydrogen site location: inferred from neighbouring sites
Least-squares matrix: full	H-atom parameters constrained
*R*[*F*^2^ > 2σ(*F*^2^)] = 0.040	*w* = 1/[σ^2^(*F*_o_^2^) + (0.0465*P*)^2^ + 0.5842*P*] where *P* = (*F*_o_^2^ + 2*F*_c_^2^)/3
*wR*(*F*^2^) = 0.103	(Δ/σ)_max_ < 0.001
*S* = 1.04	Δρ_max_ = 0.37 e Å^−^^3^
3103 reflections	Δρ_min_ = −0.29 e Å^−^^3^
248 parameters	Absolute structure: Flack *x* determined using 870 quotients [(*I*^+^)-(*I*^-^)]/[(*I*^+^)+(*I*^-^)] (Parsons *et al.*, 2013)
10 restraints	Absolute structure parameter: −0.040 (19)

### Special details

**Table:**

Geometry. All esds (except the esd in the dihedral angle between two l.s. planes) are estimated using the full covariance matrix. The cell esds are taken into account individually in the estimation of esds in distances, angles and torsion angles; correlations between esds in cell parameters are only used when they are defined by crystal symmetry. An approximate (isotropic) treatment of cell esds is used for estimating esds involving l.s. planes.
Refinement. The vinyl substituent on the benzene ring of the cation is disordered over two sites with a refined occupancy ratio of 0.542 (11):0.458 (11).

### Fractional atomic coordinates and isotropic or equivalent isotropic displacement parameters (Å^2^)

**Table:**

	*x*	*y*	*z*	*U*_iso_*/*U*_eq_	Occ. (<1)
O1	0.7129 (3)	0.3773 (2)	0.23060 (11)	0.0340 (6)	
O2	0.9047 (3)	0.2234 (2)	0.19192 (11)	0.0324 (6)	
O3	0.6241 (3)	0.1666 (2)	0.20070 (11)	0.0316 (6)	
S1	0.73918 (10)	0.26835 (7)	0.19052 (3)	0.0240 (2)	
C1	0.7098 (4)	0.3210 (3)	0.11156 (14)	0.0213 (7)	
C2	0.8167 (4)	0.4059 (3)	0.08477 (16)	0.0254 (7)	
H2	0.901987	0.439140	0.109530	0.030*	
C3	0.8000 (4)	0.4426 (3)	0.02186 (16)	0.0267 (8)	
H3	0.873464	0.501524	0.004257	0.032*	
C4	0.6771 (4)	0.3943 (3)	−0.01563 (16)	0.0259 (7)	
C41	0.6602 (5)	0.4228 (3)	−0.08413 (17)	0.0304 (8)	
H41	0.567919	0.390300	−0.104880	0.036*	
C42	0.7607 (5)	0.4885 (3)	−0.11868 (16)	0.0351 (8)	
H42A	0.854786	0.522910	−0.100025	0.042*	
H42B	0.739494	0.501740	−0.162365	0.042*	
C5	0.5676 (4)	0.3129 (3)	0.01217 (17)	0.0302 (8)	
H5	0.480488	0.281673	−0.012223	0.036*	
C6	0.5829 (4)	0.2760 (3)	0.07539 (16)	0.0283 (8)	
H6	0.506511	0.220260	0.093620	0.034*	
C7	0.3486 (4)	0.3494 (4)	0.39999 (17)	0.0282 (8)	
C8	0.2924 (5)	0.4592 (4)	0.4279 (2)	0.0391 (10)	
H8	0.270082	0.530860	0.402275	0.047*	
C9	0.2687 (6)	0.4656 (5)	0.4924 (2)	0.0577 (14)	
H9	0.228496	0.541594	0.510296	0.069*	
C10	0.3017 (5)	0.3649 (6)	0.5319 (2)	0.0600 (15)	
C101	0.2819 (10)	0.3361 (8)	0.6023 (3)	0.038 (2)	0.542 (11)
H101	0.324589	0.260969	0.620146	0.046*	0.542 (11)
C102	0.2045 (14)	0.4175 (8)	0.6374 (4)	0.066 (4)	0.542 (11)
H10A	0.162395	0.492277	0.618983	0.080*	0.542 (11)
H10B	0.190496	0.401869	0.681348	0.080*	0.542 (11)
C103	0.2724 (12)	0.4160 (9)	0.5973 (3)	0.033 (2)	0.458 (11)
H103	0.251488	0.503001	0.604321	0.040*	0.458 (11)
C104	0.2771 (11)	0.3345 (9)	0.6444 (4)	0.038 (3)	0.458 (11)
H10C	0.298320	0.247947	0.636175	0.045*	0.458 (11)
H10D	0.259356	0.362379	0.686556	0.045*	0.458 (11)
C11	0.3597 (5)	0.2564 (6)	0.5043 (2)	0.0538 (13)	
H11	0.384283	0.185948	0.530359	0.065*	
C12	0.3830 (5)	0.2473 (4)	0.43947 (19)	0.0385 (9)	
H12	0.422829	0.171040	0.421795	0.046*	
C13	0.3803 (4)	0.3451 (4)	0.32982 (17)	0.0329 (8)	
H13A	0.468168	0.284452	0.321549	0.039*	
H13B	0.417035	0.429445	0.315753	0.039*	
C14	0.2826 (5)	0.3111 (5)	0.22225 (17)	0.0528 (13)	
H14A	0.190237	0.290203	0.195519	0.079*	
H14B	0.368715	0.249929	0.214829	0.079*	
H14C	0.320828	0.396094	0.211787	0.079*	
C15	0.0986 (4)	0.3955 (3)	0.30061 (19)	0.0330 (8)	
H15A	0.010714	0.374402	0.271685	0.050*	
H15B	0.134872	0.481981	0.292401	0.050*	
H15C	0.061038	0.388818	0.344442	0.050*	
N1	0.2336 (4)	0.3069 (3)	0.29050 (13)	0.0289 (7)	
C16	0.1808 (5)	0.1749 (3)	0.3060 (2)	0.0444 (10)	
H16A	0.148219	0.170607	0.350491	0.067*	
H16B	0.269731	0.116280	0.298539	0.067*	
H16C	0.089814	0.151749	0.278940	0.067*	

### Atomic displacement parameters (Å^2^)

**Table:**

	*U*^11^	*U*^22^	*U*^33^	*U*^12^	*U*^13^	*U*^23^
O1	0.0421 (16)	0.0313 (13)	0.0287 (12)	0.0004 (12)	0.0005 (11)	−0.0081 (10)
O2	0.0250 (12)	0.0454 (14)	0.0269 (12)	0.0102 (11)	−0.0034 (10)	−0.0010 (13)
O3	0.0370 (14)	0.0303 (13)	0.0275 (13)	−0.0076 (12)	−0.0008 (11)	0.0046 (11)
S1	0.0258 (4)	0.0259 (4)	0.0203 (3)	0.0004 (4)	−0.0009 (3)	−0.0022 (3)
C1	0.0243 (17)	0.0198 (14)	0.0198 (14)	0.0036 (14)	−0.0016 (13)	−0.0010 (12)
C2	0.0218 (16)	0.0237 (16)	0.0307 (17)	−0.0006 (15)	−0.0001 (14)	−0.0042 (14)
C3	0.0259 (18)	0.0232 (16)	0.0311 (17)	−0.0003 (15)	0.0064 (15)	0.0011 (14)
C4	0.0283 (18)	0.0244 (16)	0.0249 (17)	0.0043 (15)	−0.0011 (14)	−0.0019 (14)
C41	0.035 (2)	0.0286 (18)	0.0280 (18)	0.0035 (17)	−0.0043 (16)	0.0003 (15)
C42	0.035 (2)	0.0423 (19)	0.0275 (17)	0.005 (2)	0.0014 (18)	0.0049 (15)
C5	0.0280 (18)	0.0302 (18)	0.0324 (19)	−0.0023 (16)	−0.0100 (15)	0.0016 (15)
C6	0.0283 (18)	0.0257 (17)	0.0308 (18)	−0.0034 (16)	−0.0057 (14)	0.0030 (16)
C7	0.0211 (17)	0.0344 (19)	0.0292 (18)	−0.0060 (16)	−0.0032 (14)	−0.0010 (16)
C8	0.034 (2)	0.038 (2)	0.046 (2)	0.0006 (18)	−0.0138 (18)	−0.0090 (18)
C9	0.034 (2)	0.088 (4)	0.051 (3)	0.008 (3)	−0.013 (2)	−0.036 (3)
C10	0.031 (2)	0.116 (5)	0.034 (2)	−0.018 (3)	−0.0053 (18)	−0.012 (3)
C101	0.045 (5)	0.032 (5)	0.038 (5)	−0.016 (4)	−0.016 (4)	0.009 (4)
C102	0.126 (11)	0.043 (5)	0.030 (5)	−0.009 (6)	−0.009 (6)	0.005 (4)
C103	0.040 (5)	0.031 (5)	0.028 (5)	−0.003 (5)	−0.002 (4)	−0.001 (4)
C104	0.044 (6)	0.045 (6)	0.023 (5)	−0.010 (5)	0.002 (4)	0.002 (4)
C11	0.048 (3)	0.073 (3)	0.040 (2)	−0.026 (3)	−0.0168 (19)	0.025 (2)
C12	0.036 (2)	0.033 (2)	0.046 (2)	−0.007 (2)	−0.0098 (17)	0.0038 (18)
C13	0.0223 (17)	0.042 (2)	0.0347 (19)	−0.0019 (17)	−0.0027 (15)	0.0019 (17)
C14	0.039 (2)	0.094 (4)	0.0250 (19)	0.000 (3)	0.0010 (17)	−0.007 (2)
C15	0.0293 (18)	0.0291 (18)	0.041 (2)	0.0064 (16)	−0.0063 (17)	−0.0055 (17)
N1	0.0267 (15)	0.0331 (15)	0.0271 (14)	0.0018 (14)	−0.0027 (13)	−0.0027 (11)
C16	0.056 (2)	0.0216 (17)	0.055 (3)	−0.0022 (18)	−0.019 (2)	−0.0020 (19)

### Geometric parameters (Å, º)

**Table:**

O1—S1	1.448 (2)	C10—C101	1.527 (7)
O2—S1	1.460 (2)	C101—C102	1.307 (9)
O3—S1	1.459 (2)	C101—H101	0.9500
S1—C1	1.776 (3)	C102—H10A	0.9500
C1—C2	1.387 (4)	C102—H10B	0.9500
C1—C6	1.389 (5)	C103—C104	1.317 (9)
C2—C3	1.392 (5)	C103—H103	0.9500
C2—H2	0.9500	C104—H10C	0.9500
C3—C4	1.392 (5)	C104—H10D	0.9500
C3—H3	0.9500	C11—C12	1.387 (6)
C4—C5	1.386 (5)	C11—H11	0.9500
C4—C41	1.485 (5)	C12—H12	0.9500
C41—C42	1.311 (5)	C13—N1	1.533 (5)
C41—H41	0.9500	C13—H13A	0.9900
C42—H42A	0.9500	C13—H13B	0.9900
C42—H42B	0.9500	C14—N1	1.499 (4)
C5—C6	1.397 (4)	C14—H14A	0.9800
C5—H5	0.9500	C14—H14B	0.9800
C6—H6	0.9500	C14—H14C	0.9800
C7—C8	1.386 (5)	C15—N1	1.481 (4)
C7—C12	1.395 (5)	C15—H15A	0.9800
C7—C13	1.506 (5)	C15—H15B	0.9800
C8—C9	1.380 (6)	C15—H15C	0.9800
C8—H8	0.9500	N1—C16	1.501 (4)
C9—C10	1.382 (8)	C16—H16A	0.9800
C9—H9	0.9500	C16—H16B	0.9800
C10—C11	1.376 (8)	C16—H16C	0.9800
C10—C103	1.504 (7)		
			
O1—S1—O3	113.77 (15)	C10—C101—H101	120.8
O1—S1—O2	113.04 (15)	C101—C102—H10A	120.0
O3—S1—O2	112.16 (16)	C101—C102—H10B	120.0
O1—S1—C1	106.15 (14)	H10A—C102—H10B	120.0
O3—S1—C1	106.25 (15)	C104—C103—C10	116.9 (8)
O2—S1—C1	104.59 (15)	C104—C103—H103	121.5
C2—C1—C6	119.2 (3)	C10—C103—H103	121.5
C2—C1—S1	119.9 (2)	C103—C104—H10C	120.0
C6—C1—S1	120.9 (3)	C103—C104—H10D	120.0
C1—C2—C3	120.4 (3)	H10C—C104—H10D	120.0
C1—C2—H2	119.8	C10—C11—C12	121.7 (5)
C3—C2—H2	119.8	C10—C11—H11	119.1
C2—C3—C4	120.9 (3)	C12—C11—H11	119.1
C2—C3—H3	119.5	C11—C12—C7	120.5 (4)
C4—C3—H3	119.5	C11—C12—H12	119.8
C5—C4—C3	118.2 (3)	C7—C12—H12	119.8
C5—C4—C41	118.5 (3)	C7—C13—N1	113.7 (3)
C3—C4—C41	123.3 (3)	C7—C13—H13A	108.8
C42—C41—C4	126.1 (4)	N1—C13—H13A	108.8
C42—C41—H41	116.9	C7—C13—H13B	108.8
C4—C41—H41	116.9	N1—C13—H13B	108.8
C41—C42—H42A	120.0	H13A—C13—H13B	107.7
C41—C42—H42B	120.0	N1—C14—H14A	109.5
H42A—C42—H42B	120.0	N1—C14—H14B	109.5
C4—C5—C6	121.3 (3)	H14A—C14—H14B	109.5
C4—C5—H5	119.4	N1—C14—H14C	109.5
C6—C5—H5	119.4	H14A—C14—H14C	109.5
C1—C6—C5	119.9 (3)	H14B—C14—H14C	109.5
C1—C6—H6	120.0	N1—C15—H15A	109.5
C5—C6—H6	120.0	N1—C15—H15B	109.5
C8—C7—C12	117.8 (4)	H15A—C15—H15B	109.5
C8—C7—C13	120.1 (4)	N1—C15—H15C	109.5
C12—C7—C13	121.9 (4)	H15A—C15—H15C	109.5
C9—C8—C7	120.6 (4)	H15B—C15—H15C	109.5
C9—C8—H8	119.7	C15—N1—C16	109.7 (3)
C7—C8—H8	119.7	C15—N1—C14	109.1 (3)
C8—C9—C10	122.0 (5)	C16—N1—C14	108.5 (3)
C8—C9—H9	119.0	C15—N1—C13	111.1 (3)
C10—C9—H9	119.0	C16—N1—C13	111.2 (3)
C11—C10—C9	117.4 (4)	C14—N1—C13	107.2 (3)
C11—C10—C103	138.3 (6)	N1—C16—H16A	109.5
C9—C10—C103	104.1 (6)	N1—C16—H16B	109.5
C11—C10—C101	106.5 (6)	H16A—C16—H16B	109.5
C9—C10—C101	136.0 (6)	N1—C16—H16C	109.5
C102—C101—C10	118.3 (8)	H16A—C16—H16C	109.5
C102—C101—H101	120.8	H16B—C16—H16C	109.5
			
O1—S1—C1—C2	67.5 (3)	C7—C8—C9—C10	1.0 (7)
O3—S1—C1—C2	−171.1 (2)	C8—C9—C10—C11	−0.1 (7)
O2—S1—C1—C2	−52.3 (3)	C8—C9—C10—C103	175.4 (6)
O1—S1—C1—C6	−114.1 (3)	C8—C9—C10—C101	−175.7 (6)
O3—S1—C1—C6	7.3 (3)	C11—C10—C101—C102	−169.3 (7)
O2—S1—C1—C6	126.1 (3)	C9—C10—C101—C102	6.6 (12)
C6—C1—C2—C3	−1.9 (5)	C11—C10—C103—C104	−13.7 (13)
S1—C1—C2—C3	176.5 (3)	C9—C10—C103—C104	172.3 (8)
C1—C2—C3—C4	−0.7 (5)	C9—C10—C11—C12	−0.6 (7)
C2—C3—C4—C5	2.9 (5)	C103—C10—C11—C12	−174.0 (7)
C2—C3—C4—C41	−175.4 (3)	C101—C10—C11—C12	176.3 (5)
C5—C4—C41—C42	−173.8 (4)	C10—C11—C12—C7	0.2 (6)
C3—C4—C41—C42	4.5 (6)	C8—C7—C12—C11	0.7 (5)
C3—C4—C5—C6	−2.5 (5)	C13—C7—C12—C11	176.9 (4)
C41—C4—C5—C6	175.9 (3)	C8—C7—C13—N1	−88.7 (4)
C2—C1—C6—C5	2.3 (5)	C12—C7—C13—N1	95.2 (4)
S1—C1—C6—C5	−176.2 (3)	C7—C13—N1—C15	59.1 (4)
C4—C5—C6—C1	−0.1 (6)	C7—C13—N1—C16	−63.3 (4)
C12—C7—C8—C9	−1.3 (6)	C7—C13—N1—C14	178.2 (3)
C13—C7—C8—C9	−177.6 (4)		

### Hydrogen-bond geometry (Å, º)

*Cg*1 is the centroid of the C1–C6 benzene ring.

**Table:**

*D*—H···*A*	*D*—H	H···*A*	*D*···*A*	*D*—H···*A*
C14—H14*B*···O3	0.98	2.32	3.264 (5)	161
C14—H14*A*···O2^i^	0.98	2.48	3.346 (5)	147
C15—H15*A*···O1^i^	0.98	2.63	3.544 (4)	155
C15—H15*A*···O2^i^	0.98	2.49	3.348 (4)	147
C13—H13*B*···O3^ii^	0.99	2.56	3.466 (5)	152
C15—H15*B*···O2^ii^	0.98	2.60	3.477 (4)	149
C16—H16*B*···O1^iii^	0.98	2.61	3.365 (4)	134
C16—H16*C*···O2^i^	0.98	2.52	3.370 (5)	146
C41—H41···O2^iv^	0.95	2.58	3.481 (4)	157
C42—H42*B*···O1^v^	0.95	2.63	3.494 (4)	151
C5—H5···*Cg*1^iv^	0.95	2.93	3.837 (4)	161

Symmetry codes: (i) *x*−1, *y*, *z*; (ii) −*x*+1, *y*+1/2, −*z*+1/2; (iii) −*x*+1, *y*−1/2, −*z*+1/2; (iv) *x*−1/2, −*y*+1/2, −*z*; (v) −*x*+3/2, −*y*+1, *z*−1/2.
